# Orbital Lymphoma Presenting As Recurrent Orbital Cellulitis: A Diagnostic Challenge

**DOI:** 10.7759/cureus.70759

**Published:** 2024-10-03

**Authors:** Farhana Ishak, Siti Nur Baizury Hassan, Adlina Abdul Rahim

**Affiliations:** 1 Department of Ophthalmology, Hospital Sultanah Bahiyah, Alor Setar, MYS

**Keywords:** cranial osteomyelitis, culture negative orbital cellulitis, orbital cellulitis, orbital lymphoma, recurrent orbital cellulitis

## Abstract

Orbital cellulitis is an infection of the orbital tissue behind the orbital septum. We report a case of a 71-year-old Malay lady, a passive smoker for 20 years, presenting with recurrent orbital cellulitis at the same site. She initially presented with right periorbital swelling, redness, and reduced vision worsening over two weeks, along with a firm swelling over the right temple. CT of the brain and orbit revealed a homogenous mass extending from the right sphenoid bone to the right orbit. The initial diagnosis was right sphenoid meningioma or metastasis. Her symptoms improved after 10 days of intravenous cefuroxime, prescribed for catheter-related phlebitis over her right hand, which developed during the admission. A follow-up MRI of the brain and orbit showed osteomyelitis changes in the right orbit and sphenoid bone. Histopathology revealed chronic inflammation without malignancy, and cultures were negative. The diagnosis was revised to right orbital cellulitis secondary to cranial osteomyelitis.

The patient was lost to follow-up but returned three months later with recurrent symptoms, including right periorbital swelling, reduced vision, ophthalmoplegia, and right forehead swelling. She was treated with intravenous ceftriaxone, which resulted in partial symptom resolution. Neurosurgery planned a right craniotomy, but she was undecided and again lost to follow-up due to deteriorating health. Over time, her condition worsened, leading to readmission. A repeated CT scan of the brain and orbit showed a lobulated, enhancing soft tissue lesion in the right periorbital area with intralesional calcification and bony erosion. A biopsy confirmed it as high-grade B-cell lymphoma. The patient succumbed to the illness a few weeks later.

This case highlights that orbital lymphoma can manifest as orbital cellulitis. Failure to respond to conventional orbital cellulitis treatment should raise suspicion of a more serious underlying cause. We advocate that clinicians consider orbital lymphoma as a potential diagnosis in elderly patients presenting with recurrent, culture-negative orbital cellulitis.

## Introduction

Orbital lymphoma is a type of non-Hodgkin lymphoma that involves ocular structures, including the conjunctiva, lacrimal gland, soft tissues of the eyelid, and extraocular muscles [1-3]. Primary orbital lymphoma arises from the ocular tissues, while lymphoma originating from extra orbital sites that metastasize to the orbit is known as secondary orbital lymphoma [1]. Orbital lymphoma has been reported to affect patients aged 15 to 70 years, most commonly affecting the elderly in their seventh decade, and shows no gender predominance [1].

Orbital lymphomas typically present with a salmon-patch appearance of the conjunctiva, proptosis, a slow-growing palpable mass, or painless swelling of the eyelids [1]. Orbital lymphoma can also mimic orbital cellulitis [4-6], possibly due to direct tumor invasion causing inflammation of the orbital soft tissues [4]. This similarity can result in diagnostic challenges and may delay the initiation of appropriate treatment.

## Case presentation

**Figure 1:**
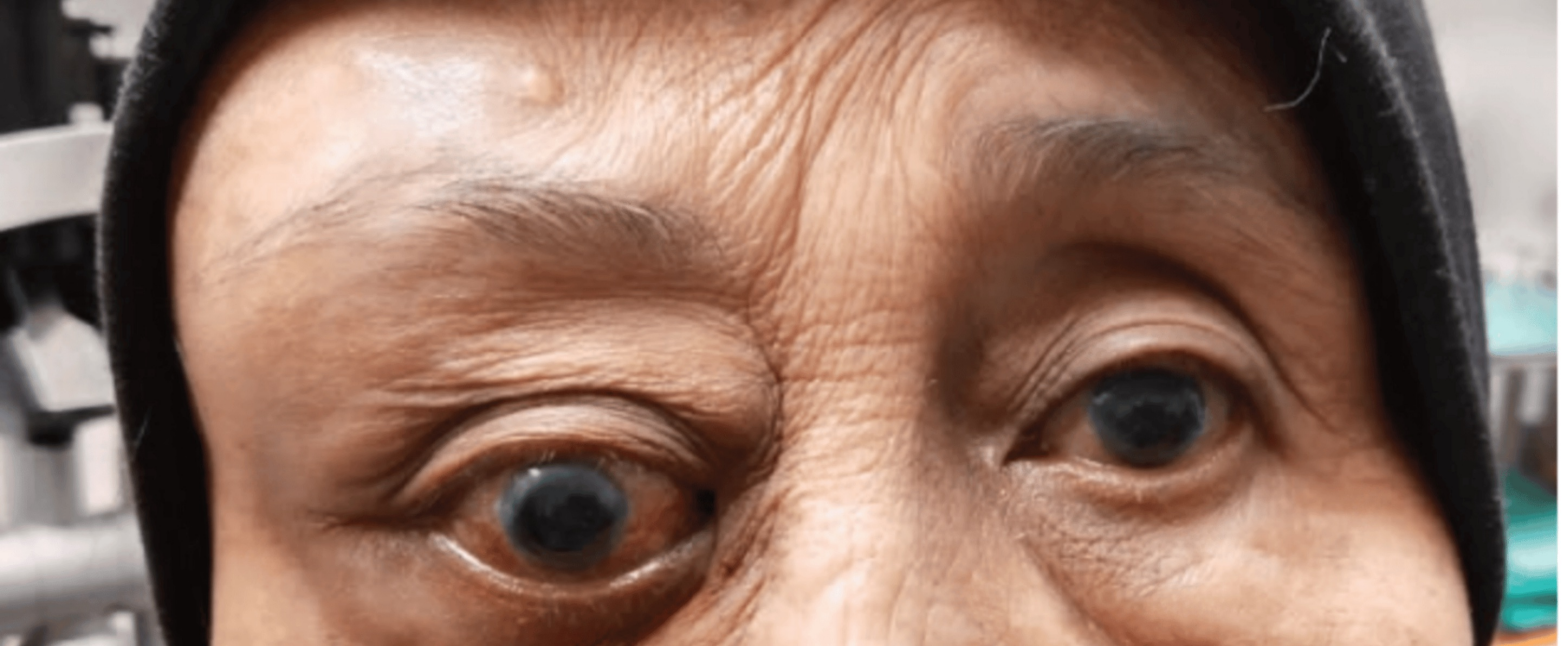
Patient on the first presentation

A 71-year-old Malay lady with hypertension, renal failure, essential thrombocytosis, and a history of ischemic stroke presented with a two-week history of right eye swelling, redness, and gradual vision loss. A passive smoker for over 20 years, she had no fever, constitutional or pulmonary symptoms, and no history of trauma. Ocular examination of the right eye showed counting finger vision, a positive relative afferent pupillary defect, non-axial proptosis with displacement of the globe downward, and ophthalmoplegia. There was a firm, non-fluctuant, non-tender swelling on the right temple measuring 6 cm x 5 cm (Figure 1). Intraocular pressure was normal. Fundus examination revealed choroidal striation at the posterior pole, a flat retina, and no signs of posterior uveitis. The left eye vision was 6/12 with an immature cataract and normal anterior and posterior segments. The systemic examination was unremarkable.

**Figure 2:**
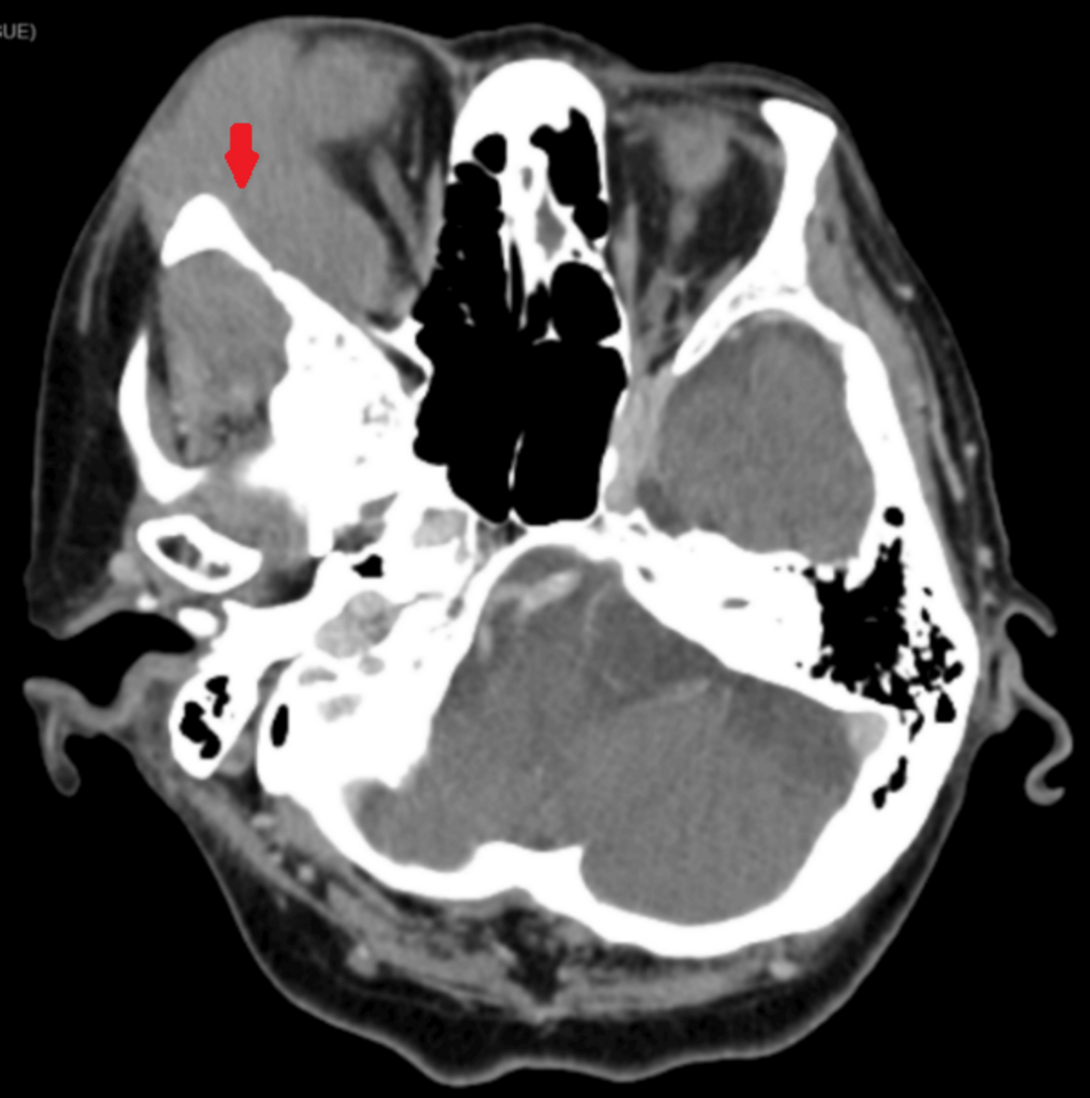
CT brain and orbit (axial view) on the first presentation CT: computed tomography

CT of the brain and orbit showed a homogenous exophytic mass arising from the greater wing of the right sphenoid bone extending to the right orbital cavity with surrounding bony erosion (Figure 2). CT of the thorax showed consolidation over the right hilar region. At that point, our working diagnosis was right sphenoid meningioma or metastasis with extension to the right orbit. She was referred to a chest physician. Bronchoscopy was performed, and lung malignancy as well as infection were ruled out.

An incisional biopsy of the right temple swelling revealed a pale, thick but fragile bone; however, no abscess or necrotic tissue was seen. Histopathology revealed chronic inflammation without a sign of malignancy, and cultures from both tissue and blood were negative.

**Figure 3:**
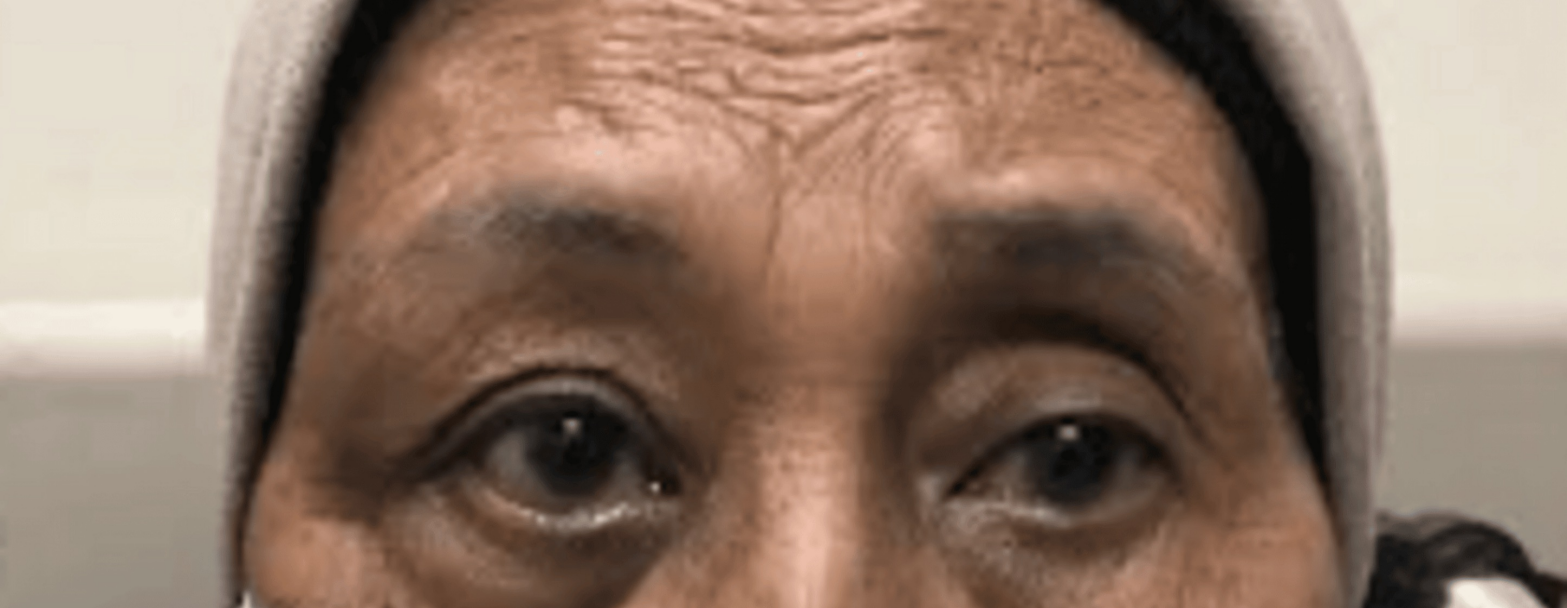
Resolved swelling after the first presentation

While in the ward, she developed right-hand cellulitis and was initiated intravenous cefuroxime 750 mg eight hourly for 10 days. Her right temple swelling then resolved, her visual acuity improved to 6/9 with resolved choroidal striation, and she regained full extraocular motility (Figure 3).

**Figure 4:**
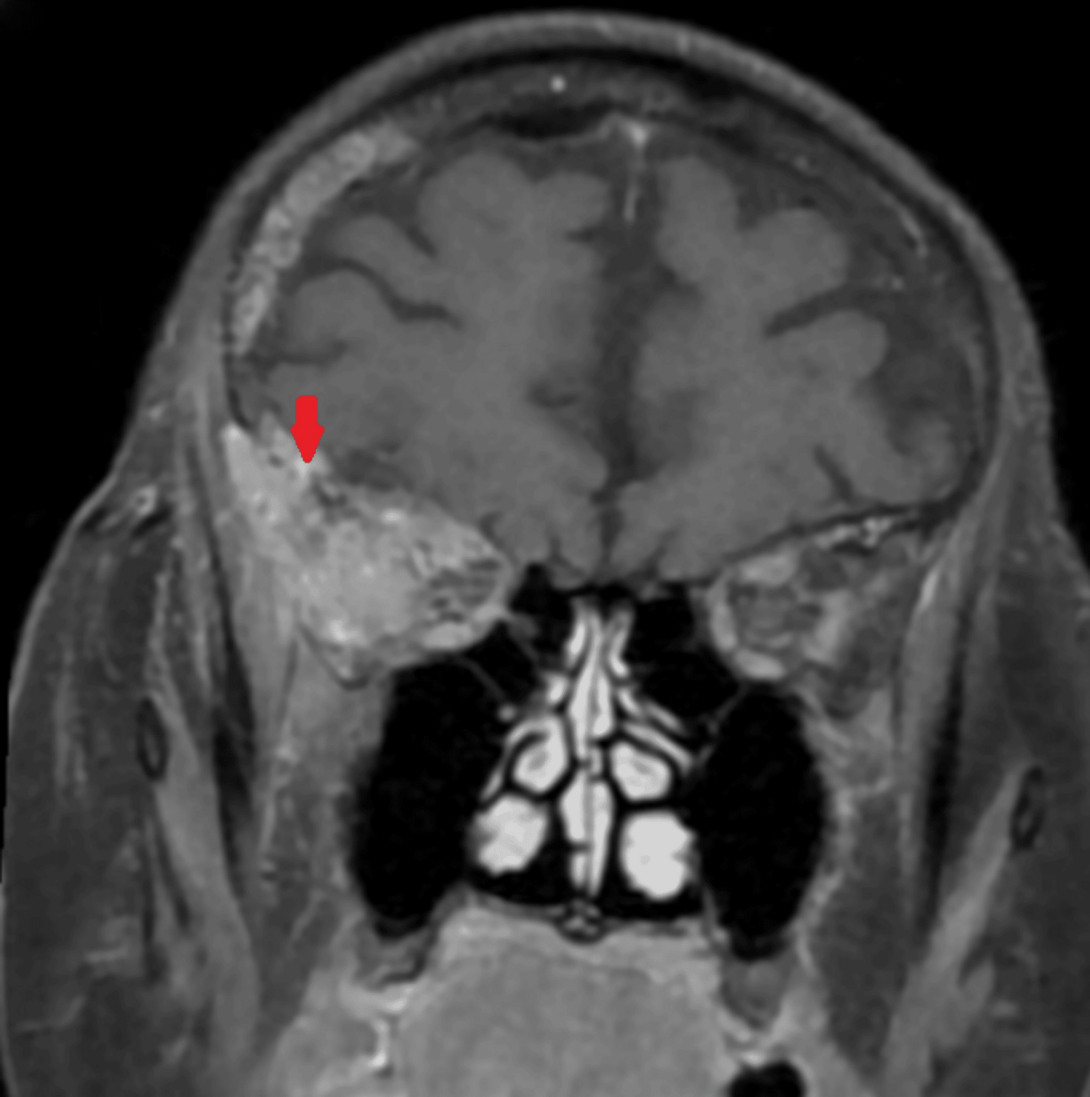
MRI orbit and brain (coronal view) on the first presentation MRI: magnetic resonance imaging

MRI of the brain and orbit performed revealed a soft tissue mass over the right orbit with bony erosion and necrotic component, consistent with osteomyelitis (Figure 4). The diagnosis was revised to right orbital cellulitis secondary to cranial osteomyelitis.

**Figure 5:**
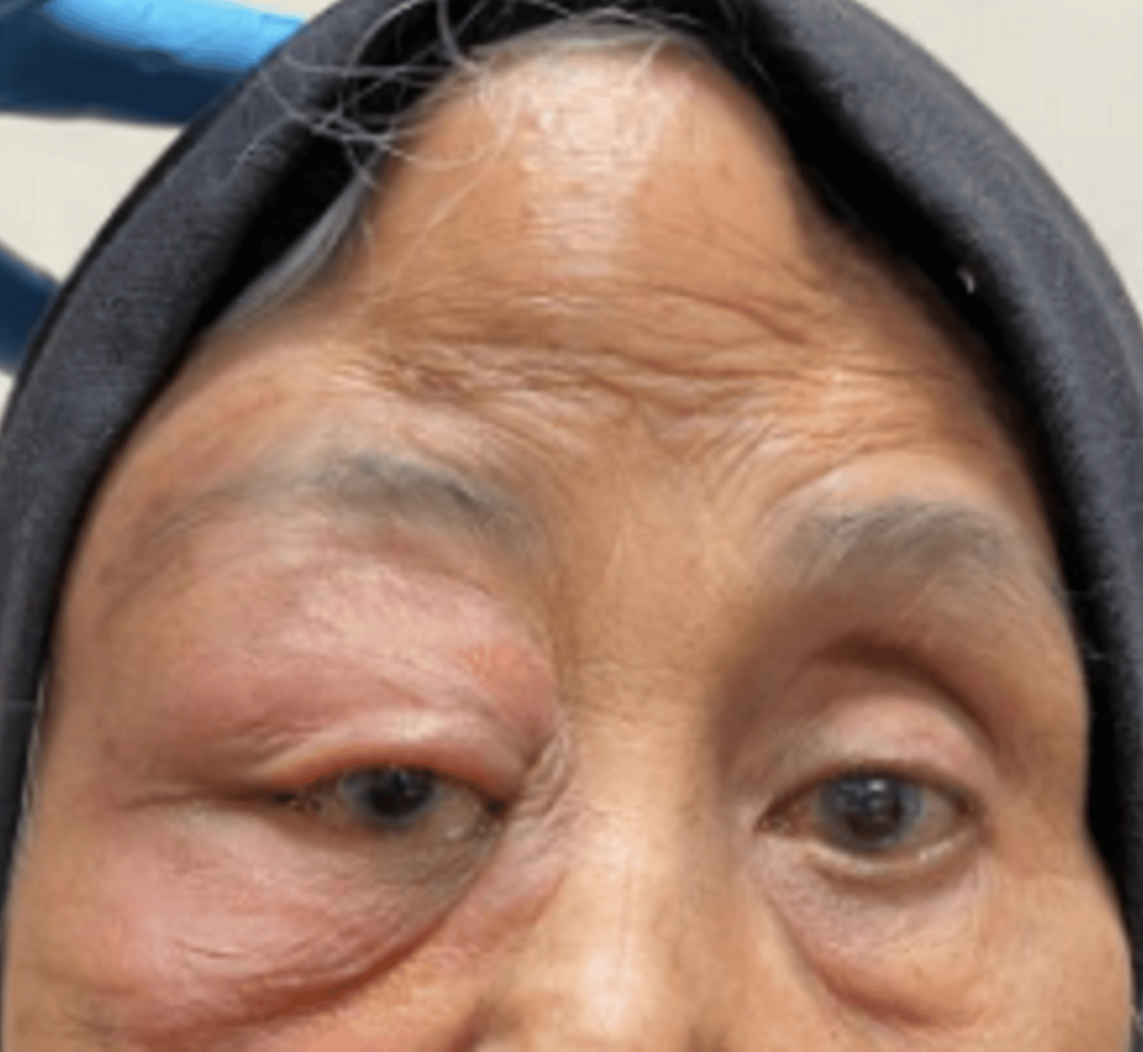
Patient on the second presentation

Three months after defaulting on follow-up, the patient re-presented with painful right eye swelling for two weeks with associated forehead swelling. Examination revealed erythematous and edematous skin over the right periorbital area, right eye proptosis, and ophthalmoplegia. Her visual acuity had decreased to 6/18, with a positive relative afferent pupillary defect. Additionally, there was a firm, non-fluctuant, non-tender swelling on the right forehead, measuring approximately 6 cm x 5 cm (Figure 5). Fundus examination revealed no posterior uveitis changes.

**Figure 6:**
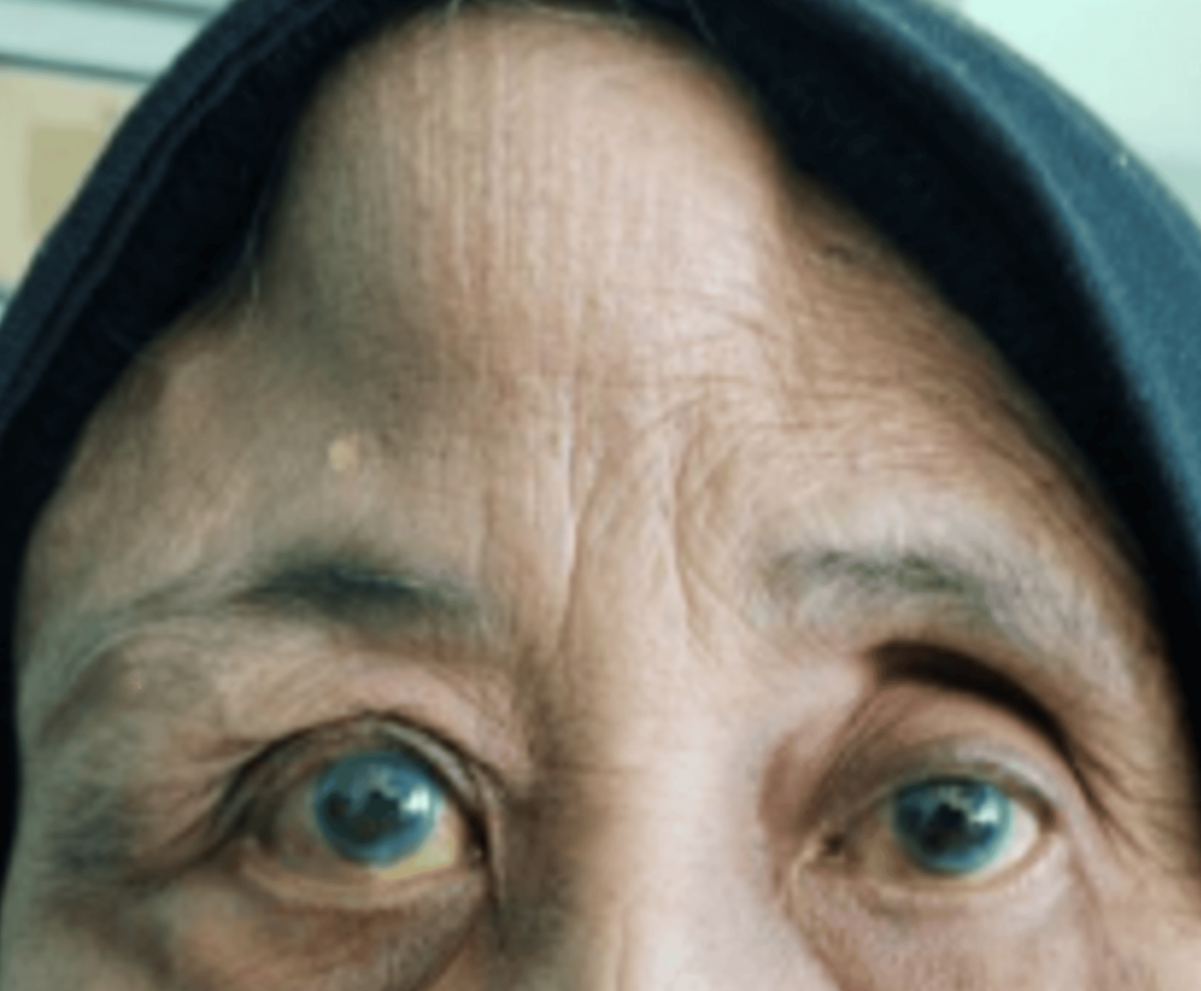
Improving right eye swelling after the second presentation

The patient was referred to both the otorhinolaryngologist and oral maxillofacial team, but no source of infection was identified. Bedside tapping of the forehead lesion did not yield any pus or fluid. Neurosurgery recommended a right craniotomy, but the patient remained undecided. After a two-week course of intravenous ceftriaxone (1 g every 12 hours), her condition partially improved: visual acuity improved to 6/12, extraocular muscle movement was full, and the swelling slightly reduced to 5x5 cm (Figure 6). Unfortunately, she was lost to follow-up due to admission for end-stage renal failure requiring dialysis.

**Figure 7:**
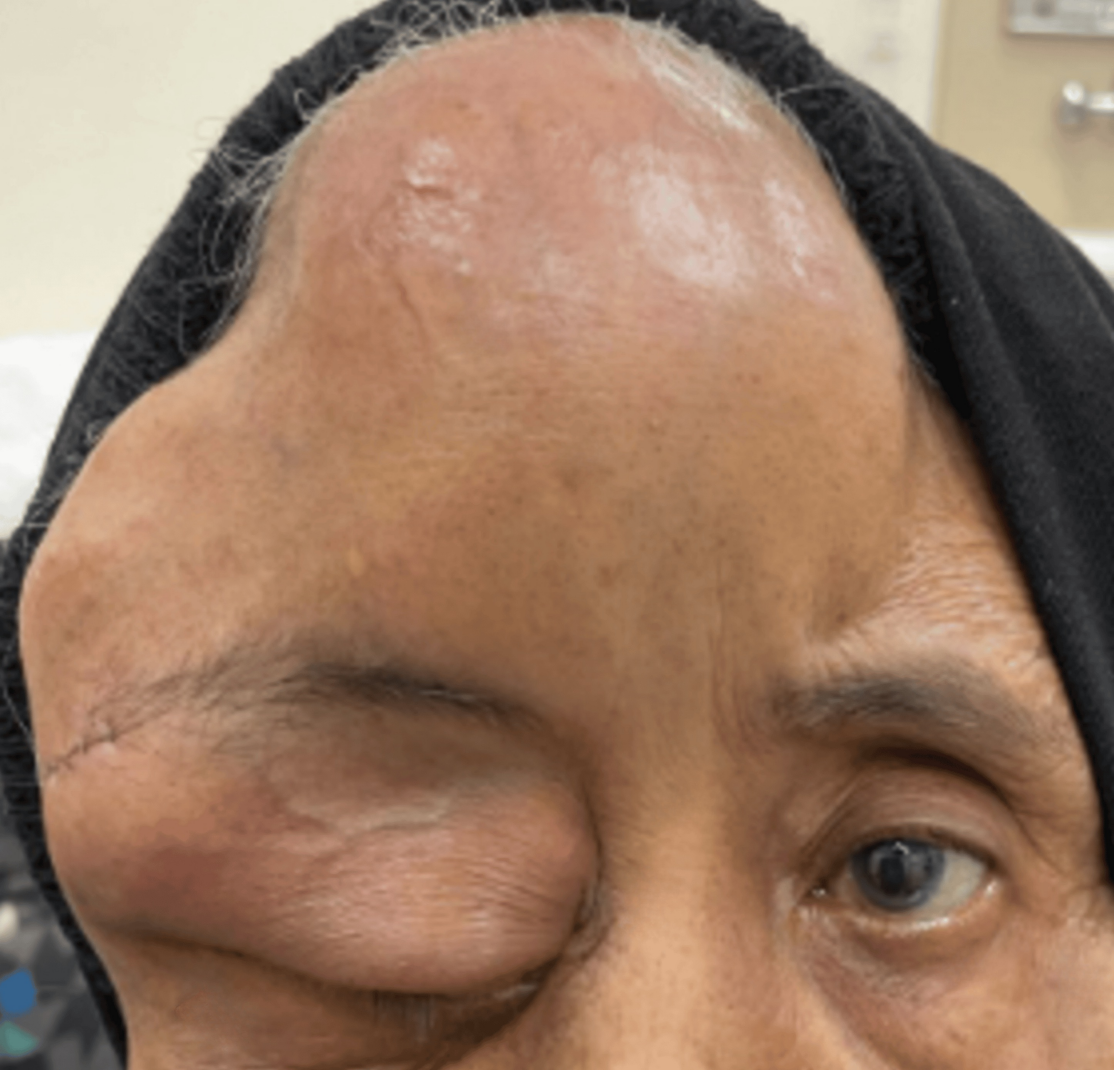
Patient during on the third presentation

Since discharged, the forehead swelling increased in size, and her vision further deteriorated to counting fingers (Figure 7). She was readmitted, and a repeat CT scan of the brain and orbit was performed.

**Figure 8:**
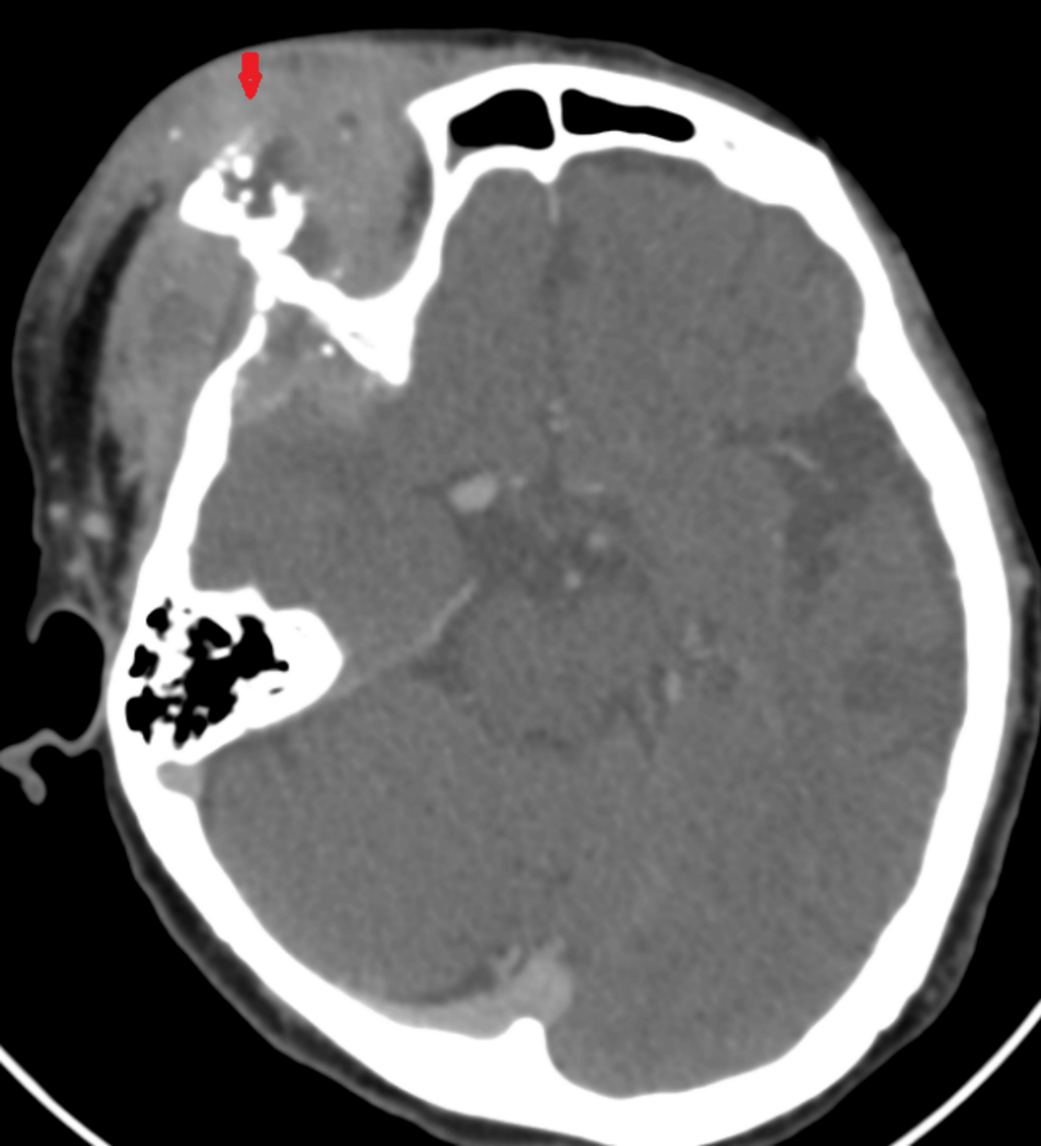
CT orbit and brain (axial view) on the third presentation CT: computed tomography

Repeated imaging revealed a lobulated, enhancing soft tissue lesion in the right periorbital area with intralesional calcification and bony erosion (Figure 8). An urgent biopsy was performed and confirmed as high-grade B-cell lymphoma. The patient succumbed to the illness a few weeks later.

## Discussion

Orbital lymphomas are predominantly B-cell in origin, with diffuse large B-cell lymphoma being the second most common non-Hodgkin lymphoma that is known for poor outcomes and higher prevalence in the elderly [2-3]. Some viruses, including HIV, Epstein-Barr, and Hepatitis C, are linked to non-Hodgkin lymphoma [3-7]. Orbital lymphoma can present unilaterally or bilaterally, with up to 20% of cases being bilateral [8].

Orbital lymphoma can mimic orbital cellulitis [4-6], possibly due to direct tumor invasion causing inflammation of the orbital soft tissues [4]. On CT of the orbit, orbital lymphoma appears as a well-defined mass and molds to the adjacent structures without bony destruction. It is homogeneous in density or slightly hyperdense compared to the extraocular muscles, with only mild to moderate enhancement after contrast. Lesions that are heterogeneous with bony destruction are suggestive of high-grade lymphomas [9].

Treatment varies by disease extent and stage. Primary orbital lymphoma is effectively treated with radiotherapy, while intermediate and high-grade lymphomas generally require chemotherapy followed by radiotherapy [8-10].

## Conclusions

We report a case of orbital lymphoma presented as recurrent orbital cellulitis with an unrepresentative initial biopsy that resulted in diagnostic delays. The patient experienced recurrent episodes of cellulitis on the same side, partially responding to intravenous antibiotics. Orbital imaging, such as CT or MRI of the brain and orbit, is essential in cases of proptosis, and a prompt biopsy should be performed if malignancy is suspected. If the initial biopsy is negative, it should be repeated to ensure adequate tissue acquisition. This case highlights the importance of considering orbital lymphoma as a diagnosis in elderly patients presenting with recurrent, culture-negative orbital cellulitis.
